# Clinical and Molecular Signatures of Gallbladder Lesions: Insights into Metabolic and Inflammatory Pathways

**DOI:** 10.3390/diagnostics16101480

**Published:** 2026-05-13

**Authors:** Andrei Bojan, Maria-Cristina Vladeanu, Catalin Pricop, Iris Bararu-Bojan, Cezar Ilie Foia, Simona Eliza Giusca, Dan Iliescu, Oana Viola Badulescu, Codruta Olimpiada Iliescu Halitchi, Maria Alexandra Martu, Amin Bazyani, Manuela Ciocoiu, Liliana Georgeta Foia

**Affiliations:** 1 Department of Surgical Specialties I, Faculty of Medicine, Grigore T. Popa University of Medicine and Pharmacy, 16 Universitatii Street, 700115 Iasi, Romania; 2 Department of Morpho Functional Sciences, Physiopathology, Faculty of Medicine, Grigore T. Popa University of Medicine and Pharmacy, 16 Universitatii Street, 700115 Iasi, Romaniamanuela.ciocoiu@umfiasi.ro (M.C.); 3 Department of Morpho Functional Sciences, Faculty of Medicine, Grigore T. Popa University of Medicine and Pharmacy, 16 Universitatii Street, 700115 Iasi, Romania; 4 Department of Internal Medicine II, Faculty of Medicine, Grigore T. Popa University of Medicine and Pharmacy, 16 Universitatii Street, 700115 Iasi, Romania; 5 Department of Pediatrics, Faculty of Medicine, Grigore T. Popa University of Medicine and Pharmacy, 16 Universitatii Street, 700115 Iasi, Romania; 6 Department of Periodontology, Faculty of Dental Medicine, Grigore T. Popa University of Medicine and Pharmacy, 16 Universitatii Street, 700115 Iasi, Romania; 7 Department of Interventional Cardiology, Heart Institute, 700503 Iasi, Romania; 8 Department of Surgery, Faculty of Dental Medicine, Grigore T. Popa University of Medicine and Pharmacy, 16 Universitatii Street, 700115 Iasi, Romania; georgeta.foia@umfiasi.ro

**Keywords:** gallbladder dysplasia, tumor markers, gallbladder cancer, proteomic analysis, inflammation

## Abstract

**Background:** Gallbladder carcinoma (GBC) represents one of the most aggressive malignancies of the hepatobiliary system, evolving along a continuum from chronic inflammation to preneoplastic lesions and invasive cancer. This progression is frequently associated with gallstones and chronic cholecystitis and shares common pathogenic mechanisms with systemic inflammatory and metabolic disorders. Despite its relatively low incidence, GBC is characterized by poor prognosis, largely due to late-stage diagnosis and limited understanding of its molecular underpinnings. **Methods:** We conducted an observational study including 60 adult patients with radiologically suspected gallbladder cancer (GBC). Patients with disseminated disease, ongoing oncologic treatment, or synchronous malignancies were excluded. Fasting venous blood samples were collected to evaluate tumor markers and biochemical parameters, including carcinoembryonic antigen (CEA) and carbohydrate antigen CA 19-9. Surgical specimens were analyzed histopathologically and staged according to the European Society for Medical Oncology TNM classification system. Statistical analysis was performed using SPSS software (version 26.0), with appropriate parametric or non-parametric tests applied based on data distribution, and a *p*-value < 0.05 considered statistically significant. **Results:** Based on histological findings, patients were stratified into benign gallbladder disease (GBD) and GBC groups. CA 19-9 demonstrated higher mean serum levels with lower variability compared to CEA, suggesting superior sensitivity and diagnostic stability for gallbladder adenocarcinoma. In contrast, CEA levels exhibited greater fluctuation, limiting its reliability as a standalone biomarker. Importantly, the combined use of CA 19-9 and CEA improved diagnostic accuracy, supporting a multimarker approach for better clinical stratification. Our findings highlight the diagnostic value of CA 19-9 as a robust biomarker in GBC and support the integration of combined biomarker panels. Beyond tumor markers, the study identified a strong interplay between systemic inflammation and metabolic comorbidities, with obesity and hypertension significantly associated with chronic gallbladder pathology, and diabetes mellitus contributing to increased risk of acute inflammatory episodes. Elevated inflammatory markers, leukocytosis, and cholestatic enzyme alterations further supported the presence of a systemic inflammatory milieu. Multivariate analysis revealed that C-reactive protein (CRP), as a marker of systemic inflammation, was significantly influenced by a combination of clinical and biochemical variables, including age, hemoglobin, hypertension, amylase, CA 19-9, and CEA, explaining over 50% of its variability and up to 85% in advanced fibrotic changes. Additionally, platelet counts were significantly reduced in adenocarcinoma and correlated specifically with CA 19-9 levels, suggesting a potential link between tumor burden, inflammation, and platelet dynamics. **Conclusions:** Therefore, the observed associations between chronic inflammation, metabolic dysregulation, and tumor marker expression suggest a potential link between gallbladder carcinogenesis and systemic cardiometabolic pathways, opening new perspectives for early detection and targeted therapeutic strategies.

## 1. Introduction

The gallbladder can be affected by a wide range of diseases, such as inflammation, preneoplastic conditions and ultimately malignant tumors. One especially aggressive type of disease is represented by gallbladder carcinoma, being well recognized that chronic inflammation of the gallbladder can result in preneoplastic alterations that may eventually progress to malignant changes. Despite its low incidence, gallbladder cancer has comparatively poor prognosis. The exact cause of gallbladder cancer is unknown. It has been claimed that the coexistence of predisposing factors and chronic inflammation plays an important role in individuals who are genetically predisposed [1,2].

Early detection of gallbladder cancer (GBC) is essential to improving patient prognosis and survival. Unfortunately, most cases are diagnosed at an advanced stage, where the tumor has already metastasized, rendering complete surgical resection either highly challenging or impossible [1,2]. Gallstones and chronic cholecystitis are implicated in 60–90% of GBC cases, reflecting their strong association with malignant transformation. Additionally, xanthogranulomatous cholecystitis (XGC) may be linked to up to one-third of GBC cases; however, the precise nature of this relationship remains inadequately understood, warranting further investigation.

Over the past three decades, little progress has been made in the early diagnosis and effective management of GBC. This stagnation is largely due to limited knowledge of the molecular and cellular mechanisms underlying its pathogenesis. Although only a minority of gallbladder lesions are malignant, chronic inflammation—especially from untreated chronic cholecystitis—can trigger a cascade of premalignant changes that may eventually culminate in invasive carcinoma. Recent studies have emphasized the importance of precursor lesions, particularly in the context of apomucin expression in gallbladder epithelium, as critical early events in carcinogenesis [3,4]. Incidental detection of gallbladder cancer during cholecystectomy for gallstones presents a vital opportunity for timely intervention. Because advanced-stage diagnoses are often accompanied by lymph node involvement and increased risk of recurrence after surgery, such incidental findings underscore the need for improved diagnostic vigilance and biomarker screening [5,6]. In this regard, tumor markers such as carcinoembryonic antigen, CA 125, AFP and CA 19-9 hold significant promise. These biomarkers, when interpreted in conjunction with clinical and histopathological data, may enhance early diagnostic accuracy and help differentiate between benign and malignant gallbladder lesions. To further elucidate the complex pathophysiology of GBC, it is crucial to explore not only mucin-related immunohistochemical patterns but also metabolic and environmental contributors. Investigations into genetic abnormalities—such as chromosomal aberrations and mitotic irregularities in the gallbladder mucosa—may offer valuable insights into carcinogenic pathways [7,8].

Tumor markers such as carcinoembryonic antigen (CEA), CA 125, AFP (Alpha-fetoprotein) and CA 19-9 are examples of lab parameters frequently used in the identification of various malignancies. Although they are not highly sensitive, CEA and CA 19-9 have historically been employed as tumor markers for gallbladder cancer (GBC). When these signs are used alone to diagnose GBC, the results are sometimes inconsistent due to their reduced sensitivity. While using CEA or CA 19-9 alone can yield some diagnostic information, the outcomes are not always accurate. The combination of these tumor indicators has been acknowledged to be a promising tool for improving the specificity and sensitivity of GBC diagnosis; however, only a small number of studies have assessed the combined use of these markers in GBC diagnosis. The accuracy of GBC diagnosis has improved significantly as a result of the mixed use of markers [9,10,11].

The aim of this study was to evaluate whether the combined analysis of tumor marker levels and Inflammatory patterns enhances the accuracy of diagnosing gallbladder lesions, including gallbladder cancer.

## 2. Materials and Methods

We conducted a retrospective observational study from October 2019 to August 2023, at the General Surgery Clinic and Department of Biochemistry affiliated to the University of Medicine and Pharmacy “Grigore T. Popa” from Iasi. The institutional ethics committees of the hospital and university approved the study no. 533/6 February 2025, and the protocols followed the principles outlined in the Helsinki Declaration. The period from January 2019 to December 2023 was selected based on the availability of complete medical records and consistent diagnostic protocols during this timeframe, ensuring reliable and comparable data for retrospective analysis.

**Figure 1:**
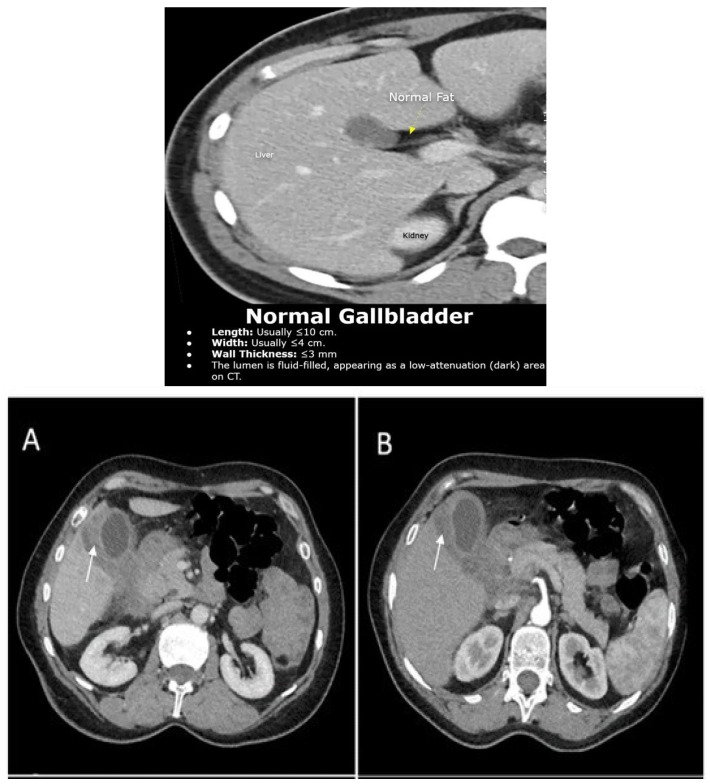
Contrast-Enhanced CT Imaging of the Gallbladder Left: Normal gallbladder aspect (**Upper**). Panels (**A**,**B**): Gallbladder with irregular mural thickening (white arrow), associated pericholecystic fluid, and loco-regional inflammatory lymphadenopathy.

Inclusion criteria: Patients aged 18 years or older were included if they were highly suspected of having gallbladder cancer (GBC) based on combined clinical and radiological findings. Clinical suspicion was raised in the presence of symptoms such as persistent right upper quadrant pain, jaundice, unexplained weight loss, anorexia, or constitutional symptoms. The radiological findings were obtained by institutional radiologists using abdominal ultrasonography (USG) and/or contrast-enhanced computed tomography (CT). Suspicion was raised by features such as irregular gallbladder wall thickening, an intraluminal mass, gallbladder polyps, or a porcelain gallbladder—Figure 1. Gallbladder polyps measuring ≥ 10 mm in diameter on imaging were considered a suspicious radiological finding and were included as part of the selection criteria for patients at risk of gallbladder cancer. This threshold is supported by existing guidelines, which associate polyps ≥ 10 mm with a higher risk of malignancy and recommend cholecystectomy in such cases. Exclusion criteria: Patients with disseminated GBC, those undergoing radiation or chemotherapy or experiencing a simultaneous second primary malignancy.

Age, weight, BMI (body mass index) and a number of biochemical markers were recorded and analyzed, along with liver and kidney function tests, full blood counts and tumour markers (AFP, CEA and CA 19-9) and inflammatory parameters. Following surgical procedures for each patient, a histopathological examination (HPE) was performed on the specimens. For cancer staging, the TNM staging system was used. Patients were classified into the benign gallbladder disease (GBD) group or the GBC group based on the results of the HPE test.

### 2.1. Preoperative Workup

Every patient had a comprehensive clinical assessment, as well as imaging scans, blood tests and tumor markers evaluation. The routine assessments included a chest X-ray, abdominal ultrasonography with Doppler and a contrast-enhanced CT scan (CECT). Selective upper gastrointestinal endoscopy was performed on patients suspected of having gastro-duodenal infiltration. Using an electrochemiluminescence immunoassay, the levels of serum CEA and CA19-9, CA 125, AFP were measured; the normal values were as follows: CEA: 0–4 ng/mL, CA 19-9: 0–4 U/mL, CA 125: 0–35 U/mL, and AFP (Alpha-fetoprotein): 0–7 ng/mL.

### 2.2. Perioperative Strategy

**Figure 2:**
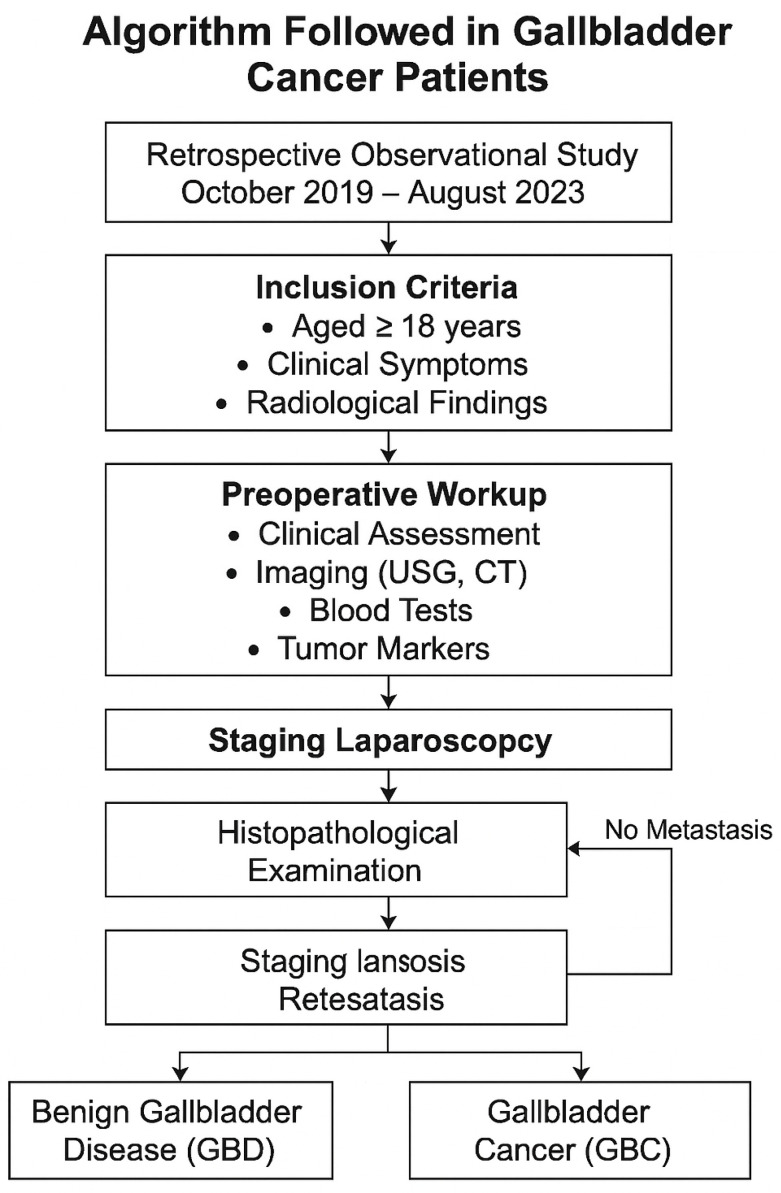
Diagnostic and Management Algorithm for Suspected Gallbladder Cancer Patients.

For final treatment, patients deemed to have a treatable condition following the outpatient evaluation process were admitted. First, a staging laparoscopy was performed on each patient to inspect eventual signs of metastasis. A biopsy could confirm metastases, in which case the final surgical procedure was cancelled—Figure 2.

### 2.3. Statistical Analysis

Statistical analysis was performed using SPSS software (version 26.0). Quantitative data were expressed as mean ± standard deviation or median with interquartile range, depending on data distribution. The choice of statistical tests was based on the distribution characteristics of the variables. Continuous variables were compared using Student’s *t*-test for parametric data or the Mann–Whitney U test for non-parametric data. Categorical variables were analyzed using the chi-square test or Fisher’s exact test, as appropriate. Multivariate regression analysis was performed to assess the relationship between C-reactive protein (CRP) and selected clinical and biochemical parameters. A *p*-value < 0.05 was considered statistically significant.

#### Gallbladder Cancer Surgery

Surgical resection remains the cornerstone of curative treatment for gallbladder cancer (GBC), with the extent of surgery tailored to tumor stage and pathological risk factors. For early-stage tumors (T1a), confined to the lamina propria, simple cholecystectomy is considered adequate due to the negligible risk of lymphatic dissemination. In contrast, the optimal management of T1b tumors, which invade the muscular layer, remains a subject of ongoing debate. While some authors report acceptable outcomes with simple cholecystectomy, current evidence and most international guidelines favor an extended cholecystectomy, given the increased risk of lymphovascular invasion and regional lymph node metastasis, reported in up to 15–20% of cases. Therefore, hepatic resection of segments IVb and V combined with regional lymphadenectomy is generally recommended to achieve oncological clearance and accurate staging.

For T2 tumors, which invade the perimuscular connective tissue, extended cholecystectomy with systematic lymphadenectomy is considered mandatory. The prognostic impact of lymph node involvement is particularly significant in this stage, as nodal metastases represent one of the strongest predictors of survival. Standard lymphadenectomy includes removal of cystic duct, pericholedochal, periportal, and hepatic artery lymph nodes, allowing for both therapeutic benefit and precise staging according to current TNM classifications. The distinction between N1 (limited regional involvement) and N2 disease has important prognostic and therapeutic implications, influencing both surgical strategy and the need for adjuvant therapy.

In our cohort, patients without evidence of distant metastasis on staging laparoscopy proceeded to definitive surgical management, with the extent of resection guided by intraoperative findings and histopathological assessment. Radical resection, including adequate lymph node dissection, offers the best chance for long-term survival; however, prognosis remains significantly poorer in patients with nodal involvement or locally advanced disease, underscoring the critical importance of accurate staging and appropriate surgical planning.

## 3. Results

The analysis of personal medical history revealed a high prevalence of cardiometabolic comorbidities within the study cohort. Obesity was present in 60% of patients and was significantly more frequent among women compared to men. Additionally, obesity was more prevalent in patients under 65 years of age, suggesting an age- and sex-related susceptibility pattern. Hypertension was identified in more than half of patients and was also more common among women, although without strong statistical. A higher prevalence was observed in patients older than 65 years, indicating a trend toward increased cardiovascular burden with aging. These findings highlight a clustering of metabolic and cardiovascular risk factors within the studied population, suggesting that gallbladder pathology may be closely linked to systemic cardiometabolic dysfunction. Obesity, in particular, plays a central role in this interaction, as it promotes chronic low-grade inflammation, insulin resistance, and dyslipidemia—mechanisms that are also fundamental in the development of atherosclerosis. The coexistence of obesity, hypertension, and diabetes reflects the pathophysiological framework of metabolic syndrome, which may contribute not only to cardiovascular morbidity but also to alterations in biliary composition, gallbladder motility, and inflammatory responses. Consequently, these data support the concept that gallbladder disease should be considered within the broader context of systemic metabolic and cardiovascular disorders, rather than as an isolated condition.

The analysis of demographic and clinical characteristics revealed a significantly higher prevalence of gallbladder pathology among female patients (75%, *p* = 0.001), suggesting increased susceptibility in this subgroup. Notably, type 2 Diabetes Mellitus had a high incidence in the analyzed subgroups, indicating a potential age-dependent metabolic phenotype within the cohort. In contrast, COVID-19 infection was observed in 6.7% of patients, predominantly affecting individuals over 65 years (*p* = 0.033), highlighting increased vulnerability in the elderly population. Risk stratification demonstrated that key cardiometabolic comorbidities significantly influenced the development of chronic cholecystitis: patients with obesity exhibited a more than ninefold increased risk (RR = 9.11; 95% CI: 3.05–27.20; *p* = 0.001), while hypertension was associated with an approximately fivefold increase (RR = 4.84; 95% CI: 2.42–9.68; *p* = 0.001), supporting the role of systemic inflammation and metabolic dysregulation in biliary disease pathogenesis. Biochemical analysis further revealed significant alterations in patients with chronic cholecystitis, including elevated liver enzymes (AST and ALT), increased serum sodium levels, and markedly higher amylase values, alongside reduced hemoglobin and hematocrit levels, reflecting a combination of hepatic, pancreatic, and systemic involvement. Tumor markers demonstrated distinct patterns, with CA19-9 and CA-125 levels being higher in patients without chronic cholecystitis, suggesting differing biological profiles between inflammatory and neoplastic conditions. Collectively, these findings support the concept that gallbladder disease is linked to systemic cardiometabolic dysfunction, sharing some common pathophysiological mechanisms with conditions such as metabolic syndrome, including chronic low-grade inflammation, oxidative stress, and altered metabolic homeostasis. Risk stratification demonstrated that metabolic and infectious factors significantly influenced the occurrence of acute cholecystitis: diabetes mellitus was associated with a 3.44-fold increased risk (RR = 3.44; 95% CI: 2.47–4.79; *p* = 0.009), likely mediated by metabolic dysregulation, microvascular impairment, and increased susceptibility to inflammation and infection, while prior SARS-CoV-2 infection was associated with a 2.24-fold increased risk, suggesting a potential contribution of systemic inflammation and endothelial dysfunction, although without strong statistical significance. Laboratory findings further emphasized distinct inflammatory and biochemical profiles: acute cholecystitis was characterized by significant leukocytosis (11,312 vs. 8695/µL, *p* = 0.002) and elevated cholestatic markers such as alkaline phosphatase (134.0 vs. 100.81 U/L, *p* = 0.006), along with markedly increased tumor-associated biomarkers, including CA19-9 (187.01 vs. 24.32 ng/mL, *p* = 0.001), CEA (9.54 vs. 2.36 ng/mL, *p* = 0.001), AFP (8.03 vs. 4.64 ng/mL, *p* = 0.001), and CA-125 (198.93 vs. 23.59 ng/mL, *p* = 0.001), reflecting an intense inflammatory response and hepatobiliary stress. Furthermore, multivariate regression analysis demonstrated that systemic inflammation, as reflected by C-reactive protein (CRP), is strongly influenced by a combination of clinical and biochemical variables, including age, hemoglobin, hypertension, amylase, CA19-9, and CEA, explaining over 50% of CRP variability in adenocarcinoma, metaplasia, and chronic cholecystitis, and up to 85% in cases with parietal fibrosis. Collectively, these findings highlight that gallbladder disease may represent not only a localized pathology but also a manifestation of systemic inflammation.

Out of the total number of 60 patients participating in our study, 5% were identified with adenocarcinoma, 8.3% of patients had metaplastic lesions. The remaining patients were diagnosed with benign gallbladder disease (GBD) or dysplasia. The median age of the cohort was 64 years (interquartile range: 28–85), and the study population was predominantly female (Table 1).

Tumor markers such as CA 19-9 and CEA have been found to be significantly elevated in patients with gallbladder cancer (GBC). In addition, the mean level of CA 19-9 was higher than that of CEA, indicating that CA 19-9 is generally more elevated in adenocarcinoma cases. Furthermore, CA 19-9 levels exhibited a narrower range compared to CEA, suggesting a less variability of CA 19-9 levels among patients. In contrast, the standard deviation of CEA levels was higher, reflecting greater variability in CEA levels among the same patient group (Table 2). This analysis points out that CA 19-9 not only has a higher average level but also displays less fluctuation compared to CEA, emphasizing its potential as a more consistent biomarker for adenocarcinoma.

In patients diagnosed with adenocarcinoma, multivariate analysis revealed a significant correlation between independent variables and CA19-9 tumor marker levels, indicating that clinical and biochemical factors substantially influence its concentration.

Age and systemic inflammation, reflected by CRP levels, were found to be key determinants of CA19-9 levels, explaining 45% of its variability (*p* = 0.001). The inclusion of carcinoembryonic antigen (CEA) further strengthened this relationship, increasing the explained variability to 86.9% (*p* = 0.001), highlighting a strong association between these parameters and CA19-9 levels.

When additional factors such as CA125 were considered, the proportion of explained variability reached 98.7% (*p* = 0.001), emphasizing the complex interplay between biochemical and clinical factors in determining CA19-9 concentrations. These findings reinforce the importance of tumor markers and inflammatory parameters in monitoring adenocarcinoma progression (Table 3).

When comparing the markers, we observed that adenocarcinoma was associated with more elevated CA 19-9 levels in contrast to metaplastic lesions.

In the histopathological examination of the gallbladder samples, approximately 8.3% of patients exhibited signs of metaplasia, a histological change that may indicate a predisposition to more serious conditions, including malignancies. Among the total cases of metaplasia, 66% were of pyloric metaplasia, and 34% were of osseous metaplasia.

**Figure 3:**
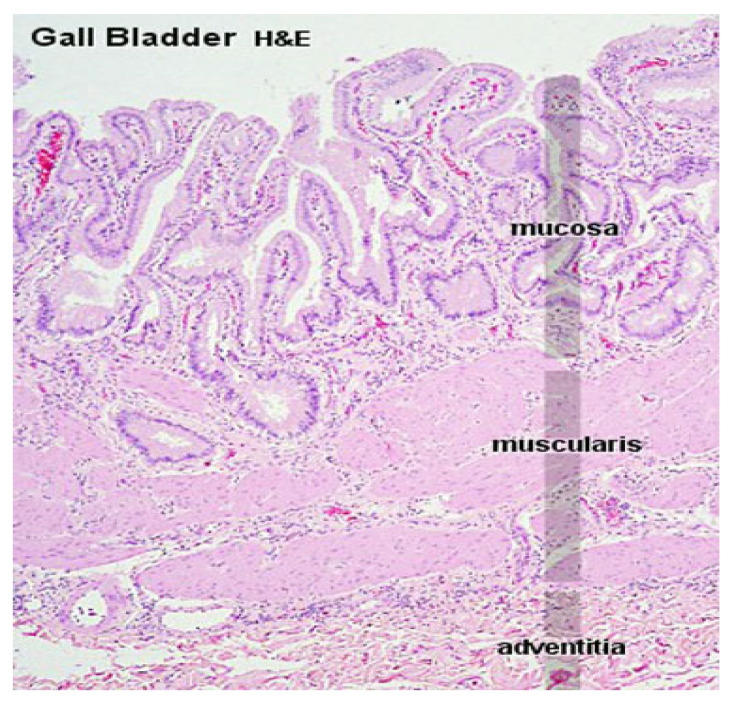

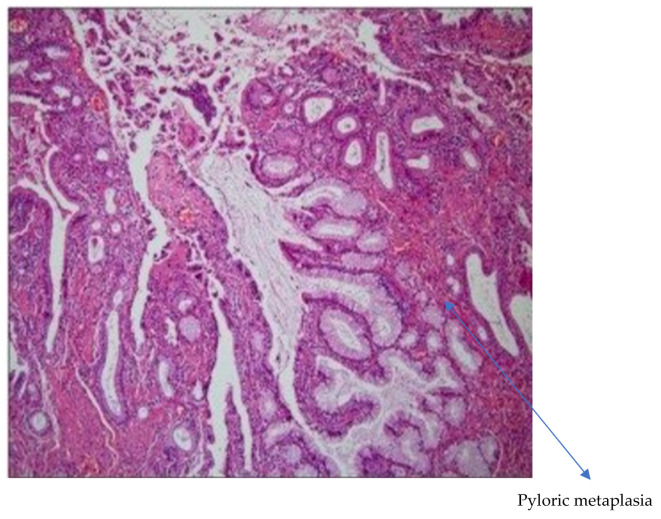
Histopathological Analysis of Gallbladder Biopsy Fragments (Hematoxylin & Eosin stain): The image shows a comparative section of gallbladder mucosa. On the left, the tissue displays normal histological features, including a uniform columnar epithelial lining, intact lamina propria, and absence of inflammation or architectural distortion. On the right, the mucosa reveals pyloric metaplasia, characterized by the replacement of native epithelium with tightly packed glands resembling those of the gastric pylorus. These glands are lined by mucin-secreting columnar cells with basally located nuclei and pale eosinophilic cytoplasm. Pyloric metaplasia is often associated with chronic inflammation and may represent a preneoplastic change in the gallbladder mucosa.

Pyloric gland metaplasia, with perineural and intraneural invasion, is an incidental microscopic finding in gallbladders with cholelithiasis and should not be confused with well-differentiated adenocarcinoma. The lobular arrangement of the glands and their cytological characteristics aid in differential diagnosis—Figure 3.

**Figure 4:**
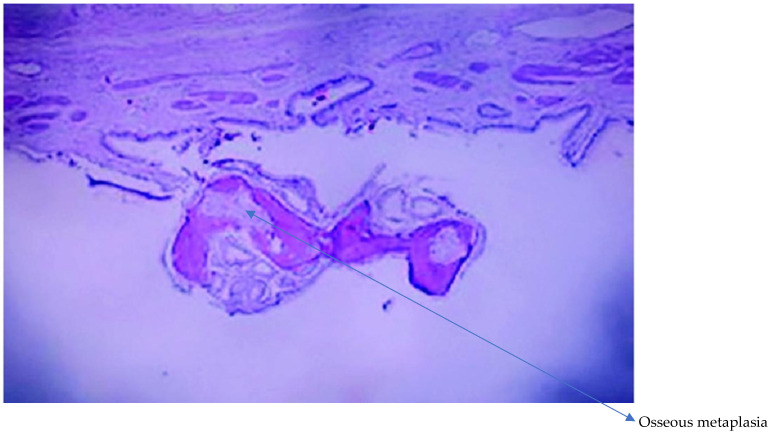
Analysis of gallbladder biopsy fragments showing osseus metaplastic features—osseous metaplasia presenting as a small polypoid lesion, identified incidentally in a mildly inflamed gallbladder (H&E stain).

Heterotopic bone metaplasia in the gallbladder mucosa is a rare histological change in which bone tissue forms abnormally within the gallbladder mucosa. It is often associated with inflammatory processes of varying intensity. This condition can present in mild forms, characterized by moderate inflammation, as well as in more severe forms, where the inflammation becomes more pronounced and may lead to damage of the gallbladder tissue Figure 4.

In our patients diagnosed with metaplasia, statistical analysis revealed a significant correlation between CA19-9 levels and multiple clinical and biochemical factors (Table 4). When considering age and C-reactive protein (CRP) levels, these two parameters accounted for 12.3% of CA19-9 variability (*p* = 0.004).

The inclusion of carcinoembryonic antigen (CEA) significantly increased the explained variability of CA19-9 levels to 98.2% (*p* = 0.001). This highlights the crucial role of CEA in the inflammatory response and metaplastic changes, suggesting that tumor markers may provide valuable insights into disease progression and severity.

Further addition of CA125, alongside age, CRP, and CEA, accounted for 99.6% of CA19-9 variability (*p* = 0.001), indicating a strong relationship between these factors. When AFP and amylase were included, the explained variability remained at 99.6% (*p* = 0.05).

Patients with gallbladder lesions have impaired inflammation patterns. The results of our analysis indicate that CRP levels in patients with adenocarcinoma are significantly influenced by multiple clinical and biochemical factors, including age, hemoglobin levels, hypertension, amylase, and tumor markers such as CA19-9 and CEA. Our statistical model demonstrated that these variables account for 53% of CRP variability (*p* = 0.004), highlighting the role of systemic inflammation in adenocarcinoma and its potential interaction with metabolic and tumor-related processes (Table 5).

For patients diagnosed with metaplasia at the histopathological examination, statistical analysis identified a significant relationship between CRP levels and a set of independent variables, including age, hemoglobin (Hb), hypertension (HT), amylase, CA19-9, and CEA. The multivariate model showed that these variables explain over 50% of the variation in CRP levels (Adjusted R^2^ = 0.501; *p* = 0.001), emphasizing their importance in inflammation associated with metaplasia (Table 6).

This study investigated the relationship between platelet (thrombocyte) counts, coagulation parameters (INR, prothrombin activity, and activated partial thromboplastin time), and tumor markers (CEA, CA19-9, CA125, and AFP) across distinct histopathological groups, including adenocarcinoma, benign lesions, and metaplastic changes. Patients diagnosed with adenocarcinoma demonstrated significantly reduced platelet counts compared to those without malignant disease (mean: 198,000/µL vs. 267,000/µL, *p* = 0.012), suggesting a potential involvement of platelet dynamics in tumor biology. Although patients with metaplasia also exhibited lower platelet levels (mean: 215,000/µL), this difference did not reach statistical significance and no meaningful differences were observed between metaplastic and benign groups, indicating that alterations in platelet count may be more specifically associated with malignant transformation rather than early or benign lesions. Notably, a moderate but statistically significant positive correlation was identified between platelet count and CA19-9 levels in the adenocarcinoma subgroup (Pearson r = 0.56, *p* = 0.02; Spearman ρ = 0.52, *p* = 0.03), suggesting a potential link between platelet activity and tumor burden or progression. In contrast, no significant associations were observed between platelet count and other tumor markers (CEA, AFP, or CA125) in this group, indicating a degree of specificity in the interaction between platelets and CA19-9. Furthermore, in patients with benign histopathological findings, correlation analyses revealed no meaningful relationships between platelet count and tumor markers, with weak correlation coefficients and non-significant *p*-values (>0.30). Similarly, the metaplasia group showed no significant correlations (r = 0.12–0.21; *p* > 0.35), supporting the notion that platelet–tumor marker interactions are limited to malignant contexts. Overall, these findings suggest that platelet alterations and their association with CA19-9 may reflect tumor-specific biological processes in adenocarcinoma, potentially related to inflammation, tumor-induced thrombopoiesis, or platelet–tumor interactions, while remaining largely absent in benign or pre-neoplastic conditions.

Histopathological evaluation revealed that inflammatory infiltration was present in almost two thirds of cases, predominantly observed in patients with chronic cholecystitis, followed by those with acute cholecystitis. Parietal fibrosis, defined as fibrotic remodeling of the gallbladder wall, was identified in 41.1% of specimens and showed a higher prevalence in chronic cholecystitis (51.4%, *p* = 0.001) compared to acute cholecystitis (37.8%, *p* = 0.162), supporting its role as a marker of long-standing inflammatory processes and structural alteration.

In patients with adenocarcinoma, systemic inflammatory status, as reflected by C-reactive protein (CRP), was significantly associated with a combination of clinical and biochemical parameters. Multivariate regression analysis demonstrated that approximately 53% of CRP variability could be explained by age, hemoglobin, hematocrit, amylase, CA19-9, and carcinoembryonic antigen (CEA), with a statistically significant model fit (adjusted R^2^ = 0.530, *p* = 0.004).

The stepwise modeling approach illustrated the progressive contribution of each variable to the predictive performance. Age alone showed a limited association with CRP levels, while the addition of hematological parameters (hemoglobin and hematocrit) provided only a modest improvement, suggesting a minor influence of anemia-related factors on systemic inflammation. A more substantial increase in explanatory power was observed following the inclusion of amylase, indicating a potential link between inflammatory status and hepatopancreatobiliary enzymatic activity.

The most pronounced enhancement in model performance occurred with the incorporation of CA19-9, highlighting a strong relationship between tumor-associated antigen expression and systemic inflammatory response. The subsequent addition of CEA further refined the model, underscoring the role of tumor burden in modulating inflammation. These findings emphasize the complex interaction between neoplastic progression and inflammatory pathways, suggesting that elevated CRP levels in adenocarcinoma reflect not only host immune activation but also tumor-driven biological processes that contribute to disease severity.

## 4. Discussion

The present findings support the concept that gallbladder pathology, particularly adenocarcinoma, should be interpreted within the broader framework of systemic inflammation and cardiometabolic dysfunction rather than as an isolated hepatobiliary condition. The high prevalence of obesity, hypertension, and diabetes within our cohort, together with their significant associations with both acute and chronic cholecystitis, reinforces the hypothesis that metabolic syndrome plays a central role in biliary disease pathogenesis. These conditions are known to promote chronic low-grade inflammation, endothelial dysfunction, and altered lipid metabolism, mechanisms that are also fundamental in the development of atherosclerosis and may similarly contribute to gallbladder mucosal injury, impaired motility, and lithogenic bile formation.

Our data further highlight the importance of systemic inflammatory burden, as reflected by CRP, which was strongly influenced by a combination of clinical and biochemical parameters, including tumor markers and metabolic variables. The ability of multivariate models to explain more than half of CRP variability and up to the majority in fibrotic changes suggests that inflammation in gallbladder disease is multifactorial, integrating tumor-related activity, hepatopancreatobiliary dysfunction, and host metabolic status. This is consistent with emerging evidence that chronic inflammation acts as a key driver of carcinogenesis, facilitating genomic instability, tumor progression, and microenvironment remodeling.

In this context, the observed elevation of CA 19-9 in parallel with inflammatory markers, as well as its correlation with platelet counts specifically in adenocarcinoma, may reflect a complex interaction between tumor burden, systemic inflammation, and hemostatic pathways. Platelets are increasingly recognized as active participants in tumor biology, contributing to tumor growth, angiogenesis, and immune evasion through the release of cytokines and growth factors. The reduced platelet counts observed in adenocarcinoma, together with their selective association with CA 19-9, suggest a potential consumption or redistribution phenomenon within the tumor microenvironment, further supporting the link between coagulation, inflammation, and malignancy.

Moreover, the marked increase in tumor-associated biomarkers and inflammatory indices in acute cholecystitis underscores the overlap between inflammatory and neoplastic signaling pathways. This overlap may complicate differential diagnosis but also provides an opportunity for integrated biomarker-based stratification. The identification of metabolic and infectious triggers, such as diabetes mellitus and prior SARS-CoV-2 infection, further emphasizes the role of systemic factors in modulating biliary inflammation, potentially through mechanisms involving immune dysregulation, endothelial injury, and microvascular impairment.

The association between systemic cardiometabolic dysfunction and gallbladder pathology, including malignant transformation, remains incompletely understood; however, our findings support a multifactorial interplay involving metabolic, inflammatory, and microvascular mechanisms. The high prevalence of obesity, hypertension, and diabetes mellitus observed in our cohort reflects a typical metabolic syndrome profile, which is increasingly recognized as a systemic pro-inflammatory and pro-oncogenic condition. Beyond its classical cardiovascular implications, metabolic syndrome is also associated with renal involvement, ranging from early endothelial dysfunction and microalbuminuria to chronic kidney disease. This renal component is particularly relevant, as kidney injury contributes to systemic inflammation, oxidative stress, and impaired metabolic clearance, thereby amplifying the pathological milieu that may favor carcinogenesis.

From a pathophysiological perspective, insulin resistance and hyperinsulinemia promote increased hepatic cholesterol secretion and bile supersaturation, predisposing to gallstone formation and chronic cholecystitis—well-established risk factors for gallbladder cancer. Simultaneously, adipose tissue dysfunction in obesity leads to the release of pro-inflammatory cytokines (e.g., IL-6, TNF-α) and adipokines, sustaining a chronic low-grade inflammatory state. This inflammatory background is further exacerbated in patients with renal impairment, where reduced clearance of uremic toxins and persistent activation of inflammatory pathways contribute to endothelial dysfunction and immune dysregulation. These mechanisms may facilitate epithelial injury, metaplastic changes, and ultimately neoplastic transformation.

Moreover, hypertension and diabetes-related microvascular damage can impair gallbladder wall perfusion and motility, promoting bile stasis and chronic inflammation. In parallel, renal dysfunction has been associated with altered lipid metabolism and increased oxidative stress, both of which may further influence biliary composition and carcinogenic pathways. The integration of these mechanisms suggests that gallbladder disease, particularly in its neoplastic spectrum, should be viewed not merely as a localized hepatobiliary condition but as part of a broader systemic disorder involving metabolic, cardiovascular, and renal axes.

The evaluation of circulating protein biomarkers further supports the concept that gallbladder disease reflects a systemic inflammatory and metabolic disorder rather than a purely localized condition. In our cohort, C-reactive protein (CRP) levels showed a wide variability (0.29–384 mg/L; mean 27.77 mg/L; median 18 mg/L), indicating heterogeneous but frequently elevated inflammatory responses in patients with gallbladder lesions. Importantly, multivariate regression analysis demonstrated that CRP levels are significantly influenced by a combination of clinical and biochemical variables, including age, hemoglobin, hypertension, amylase, and tumor markers such as CA19-9 and CEA. These factors accounted for approximately 53% of CRP variability in patients with adenocarcinoma (*p* = 0.004) and over 50% in those with metaplastic lesions (adjusted R^2^ = 0.501; *p* = 0.001). These findings suggest that CRP acts as an integrative biomarker reflecting the interplay between systemic inflammation, metabolic dysregulation, and tumor-related processes. Although an extended proteomic panel was not included in the present study, our results provide a strong rationale for the future incorporation of additional targeted circulating proteins, such as interleukin-6, tumor necrosis factor-alpha, and adipokines (leptin and adiponectin), which are known to play key roles in linking metabolic syndrome, chronic inflammation, and carcinogenesis.

Taken together, these findings suggest that gallbladder disease represents a convergence point between metabolic dysfunction, inflammation, and oncogenesis. From a clinical perspective, this supports the need for a multidimensional diagnostic approach integrating tumor markers, inflammatory indices, metabolic profiling, and coagulation parameters. Such an approach may improve early detection, refine risk stratification, and open new avenues for targeted therapeutic strategies that address not only local disease but also the underlying systemic milieu.

Our findings indicate that serum CA 19-9 levels were significantly elevated in patients with gallbladder adenocarcinoma compared to those with metaplastic lesions. This observation suggests that CA 19-9 may function as a more specific and sensitive biomarker for malignant transformation, particularly in distinguishing adenocarcinoma from non-neoplastic or preneoplastic conditions such as pyloric or intestinal metaplasia. The marked difference in CA 19-9 expression between these histological subtypes highlights its diagnostic utility and reinforces its role in the preoperative assessment of gallbladder lesions. Although CA 19-9 is not exclusively cancer-specific and may be elevated in some benign conditions, its disproportionate elevation in adenocarcinoma cases may aid in clinical decision-making when used in conjunction with imaging and other tumor markers. These findings support the inclusion of CA 19-9 in diagnostic algorithms aimed at risk stratification and early identification of malignant gallbladder disease. Distinguishing elevated CA 19-9 and CEA levels due to malignancy from those caused by inflammation, such as cholecystitis, remains a diagnostic challenge. While both markers can be mildly elevated in benign biliary disease, significantly higher and persistent elevations are more suggestive of malignancy. Proposed cut-off values to improve specificity include >37 IU/mL for CA 19-9 and >5 ng/mL for CEA, though some studies suggest raising the CA 19-9 threshold (e.g., >39–55 IU/mL) for better discrimination. Serial measurements, combined marker analysis (e.g., CA 19-9 with CA 242), and integration with imaging and clinical findings are recommended to enhance diagnostic accuracy and assess tumor resectability more reliably. Carcinoembryonic antigen (CEA) and carbohydrate antigen 19-9 (CA19-9) are well-known cancer-associated indicators that are commonly used to help with the diagnosis and prognosis of cancers inside the digestive system. These markers have proven to be valuable tools in the assessment of pancreatic, colorectal, stomach and bile duct cancers. Furthermore, assessing the levels of CEA and CA19-9 can assist in determining the degree of resectable tumors, such as gallbladder cancer (GBC) [12].

Preoperative levels of CA19-9 were found to be helpful in predicting the likelihood of tumor resectability in a trial involving 292 patients with stage I-IV GBC [13]. However, it should be noted that this study did not examine the relationship between elevated total bilirubin levels and blood concentrations of CA19-9 and CEA, which have been linked to bile duct obstruction brought on by tumors [14,15]. In cases of GBC that are in the early stages, specifically at stage 0-I, radical resection is most frequently used. Patients that undergo such type of surgery have usually good results; over 60% of them survive five years after the procedure. These results highlight the significance of utilizing tumor markers such as CEA and CA19-9, not only for diagnosis purposes but also as useful instruments for evaluating the likelihood of surgical intervention success and long-term patient survival. The accuracy of these prognostic evaluations may be further enhanced by deepening our comprehension of the ways in which other clinical parameters, such as bilirubin levels, impact these markers [16,17].

The tumor marker known as carbohydrate antigen 19-9, or CA 19-9, is crucial for the identification and monitoring of some cancers, particularly those that affect the digestive system. First identified in individuals with colorectal cancer, CA 19-9 is currently most frequently used to diagnose pancreatic, bile duct, and gallbladder cancers. Elevated levels of CA 19-9 are not specific to any of the cancer type, however there are frequently linked to gastrointestinal neoplasia, such as gallbladder and pancreatic cancers.

CA 19-9 is helpful in diagnosing malignancies; however, it has certain limits. Its specificity may be impacted by an elevation in non-cancerous illnesses such pancreatitis, liver disorders or bile duct obstruction. Nevertheless, CA 19-9 is useful for assessing tumor burden, forecasting the likelihood that a tumor would be resectable, and tracking the advancement or return of the disease following treatment. Since its sensitivity and specificity might change based on the kind and stage of the neoplasia, it is frequently employed in conjunction with other tumor markers to increase diagnostic accuracy [18,19]. In a recent study, the greatest elevated sensitivity (CA 19-9, at 85%) and specificity (CA 125, at 81.8%) were reported in 71 patients with gallbladder cancer (GBC) [20].

Additionally, Sachan et al. demonstrated that CA 19-9 had greater sensitivity and specificity (52% and 80%, respectively) than CEA (51% sensitivity and 72% specificity) in predicting cancer load in GBC patients [21].

Furthermore, Wang et al. revealed that CA 19-9 and CA 242 showed the highest increased sensitivity and specificity at 71.7% and 98.7%, respectively [10]. Serum CA 19-9, for instance, has shown strong specificity and medium sensitivity, suggesting that it may be helpful in detecting GBC. Zhou’s study confirmed that CA 19-9 offers a constant specificity and low sensitivity for GBC identification [22].

Particularly, serum CA 19-9 has demonstrated medium sensitivity and good specificity, suggesting that it may be useful in the detection of GBC. Zhou’s study indicated that CA 19-9 provides dependable specificity and a reasonable sensitivity for GBC detection [22]. But when applied separately, these tumor markers’ performance has been a little patchy, with discrepant levels of sensitivity and specificity observed in different research. It is interesting to see that the use of all these markers, increase the sensitivity of GBC diagnosis.

In line with the previous studies, our findings support the utility of CA 19-9 as a clinically relevant biomarker in the context of gallbladder cancer (GBC). We observed that CA 19-9 levels were significantly elevated in patients with adenocarcinoma compared to those with metaplastic lesions, reinforcing its role in differentiating malignant from preneoplastic conditions. This aligns with data from Sachan et al., who reported higher sensitivity and specificity for CA 19-9 compared to CEA in assessing tumor burden in GBC patients [21], and with Wang et al., who demonstrated improved diagnostic performance when CA 19-9 was used in combination with CA 242 [10]. Moreover, our study confirms prior findings that combined use of multiple markers—specifically CA 19-9 and CEA—enhances diagnostic accuracy, mirroring reports of increased sensitivity when multimarker panels were employed [20]. Although CA 19-9 alone presents moderate sensitivity and high specificity, as also noted by Zhou [22], the integration of CEA and CA 19-9 in our cohort provided better stratification between adenocarcinoma and non-malignant lesions. These findings underscore the value of adopting a multimodal biomarker strategy for more accurate preoperative assessment of gallbladder lesions, particularly in distinguishing cases warranting surgical intervention.

When both tumor markers, CA 19-9 and CEA, exceeded their respective threshold values, diagnostic sensitivity increased significantly. This observation is consistent with previous studies reporting an approximately 8.9% improvement in diagnostic accuracy and sensitivity when a combined panel of markers (CA 19-9, CA 125, and CA 242) was used. These findings suggest that the combined assessment of multiple tumor markers may substantially enhance both sensitivity and specificity in the diagnosis of gallbladder cancer, offering a more reliable approach for early detection and more accurate disease evaluation [14,23,24,25].

The multivariate analysis of the patients with adenocarcinoma included in our study revealed a strong correlation between clinical and biochemical factors and the levels of the CA19-9 tumor marker. This suggests that these factors play a significant role in determining the concentration of CA19-9 in these patients. Specifically, age and systemic inflammation, as reflected by CRP levels, were identified as key contributors to CA19-9 levels, indicating that both patient demographics and inflammatory responses are crucial in understanding variations in tumor marker levels.

The addition of carcinoembryonic antigen (CEA) further strengthened this association, showing that the interplay between these factors provides a more comprehensive explanation of CA19-9 variability. The inclusion of CA125 in the analysis revealed an even more complex relationship between clinical, biochemical, and tumor-related factors, suggesting that these markers work together to reflect the underlying disease processes more accurately.

In our patients diagnosed with metaplasia, statistical analysis revealed a significant correlation between CA19-9 levels and clinical and biochemical factors, including age and C-reactive protein (CRP). These two parameters significantly influenced CA19-9 variability, suggesting their role as important determinants in metaplastic changes.

The inclusion of carcinoembryonic antigen (CEA) further strengthened the relationship, emphasizing its role in the inflammatory response associated with metaplasia. This suggests that tumor markers like CEA can offer valuable insights into disease progression.

When CA125 was added to the model, it clarified the relationship between the factors and CA19-9 levels. Despite adding markers like AFP and amylase, the variability explained remained largely unchanged, highlighting the key role of age, CRP, CEA, and CA125 in determining CA19-9 levels. These findings underline the importance of these tumor markers in assessing also metaplasia progression.

The glycoprotein known as carcinoembryonic antigen (CEA) is a tumor marker that is mainly utilized in the identification and treatment of malignancies, especially colorectal cancer. CEA levels are normally low in healthy adults but can rise in some types of cancer. It was first found in fetal gut tissue. CEA has been investigated for its potential to identify and track gallbladder lesions, especially when gallbladder cancer (GBC) is present. Despite being more frequently linked to gastrointestinal and colorectal malignancies, CEA is a valuable marker for evaluating GBC since it might be increased in certain people. CEA can assist in distinguishing benign diseases like chronic cholecystitis from malignant tumors in gallbladder lesions. However, because CEA levels can also increase in non-cancerous situations including inflammation or biliary obstruction, its specificity in gallbladder cancer is restricted [26,27,28]. Despite these drawbacks, CEA can be used to track tumor burden, treatment response and possible recurrence following surgery when paired with other markers such CA 19-9. It also enhances the accuracy of GBC diagnosis. Given that GBC usually manifests in a late stage, when early detection using combined tumor markers should improve treatment outcomes, its role is therefore crucial [29,30,31,32,33].

Considering the current available data, it is obvious that two of the most used tumor markers for gallbladder cancer (GBC) are CA19-9 and CEA [10,12,30]. More research is necessary to determine their involvement in predicting metastasis or unresectability. Precise assessment of the serum levels of these markers can improve the accuracy of prognostic appraisal for patients. In contrast to CEA, research by Wang et al. [10] found that CA19-9 levels rose continuously as clinical stages advanced. The present data shows that, in contrast to CEA levels, which did not substantially correlate with the formation of tumors, CA19-9 levels rise with increasing tumor burden. In a study developed by Shukla et al., out of 335 patients, 80 subjects experienced jaundice and revealed interesting facts as, in 95% of cases, CA19-9 levels greater than 90 IU/mL were linked to an incurable disease [24].

In addition, the 5-year survival rate was significantly higher in patients with CEA levels ≤ 4 ng/mL (42.8%) compared to those with levels >4 ng/mL (12.5%). Similarly, patients with CA19-9 levels ≤ 37 U/mL had a 5-year survival rate of 40%, whereas none of the patients with CA19-9 levels > 37 U/mL survived beyond 5 years, even after curative surgical resection. These findings indicate that preoperative serum levels of CEA and CA19-9 may serve as valuable prognostic indicators in patients undergoing curative treatment for GBC.

Consistent with these observations, Agarwal et al. reported that patients undergoing extended cholecystectomy with preoperative CA19-9 levels < 20 U/mL achieved a 4-year survival rate of 78%, whereas those with levels > 20 U/mL had a markedly poorer prognosis, with survival limited to approximately 3 years. Furthermore, median survival was not reached in patients with CA19-9 levels < 20 U/mL, while those with elevated levels had a median survival of approximately 12 months [20,21,34].

Our findings underline that extensive inflammation, as indicated by CRP levels, is influenced by a combination of hematological, biochemical, and clinical factors in adenocarcinoma cases. Age and hemoglobin levels may reflect the body’s inflammatory response, while the presence of hypertension could indicate a potential vascular component in the inflammatory process. Additionally, the involvement of digestive enzymes and tumor markers suggests a link between inflammatory processes and possible hepatobiliary or pancreatic dysfunction in these patients.

Our results indicate that patients with metaplasia exhibit a significant systemic inflammatory response influenced by multiple factors, including hematologic status, metabolic parameters, and tumor markers. Elevated CRP levels in these patients may reflect not only local inflammation associated with metaplastic changes but also a broader pro-inflammatory state that could impact disease progression.

These findings support the need for careful monitoring of these parameters in patients with metaplasia, considering CRP’s role as a predictive inflammatory marker and its potential to indicate disease severity. The results also suggest that inflammatory processes in metaplasia may interact with specific tumor biomarkers, which could have implications for both diagnosis and clinical management.

Chronic inflammation plays a major role in gallbladder diseases, being considered one of the main mechanisms that promote cancer development. Repeated and prolonged inflammation of the gallbladder can induce changes in tissue structure, which can progress to dysplasia and eventually to malignant neoplasms. This occurs through the activation of a continuous immune response, promoting abnormal cell proliferation and inducing DNA alterations [35]. Specifically, in chronic cholecystitis, the inflammatory environment promotes the secretion of cytokines and growth factors, which can stimulate the proliferation of gallbladder epithelial cells and lead to the formation of precancerous lesions. Additionally, chronic inflammation can contribute to the creation of a tumor microenvironment, where tumor cells can survive, multiply, and invade adjacent tissues. This microenvironment is characterized by the infiltration of connective tissue with inflammatory cells, such as lymphocytes and macrophages, which release chemicals that promote angiogenesis (the formation of new blood vessels), necessary to nourish the growing tumor [36].

Metaplastic changes in the gallbladder epithelium are considered precursors to gallbladder cancer. A study by Seretis et al. [37] found that among twelve cases of metaplasia, the majority showed intestinal metaplasia, with some cases also presenting concomitant dysplasia. In contrast, Khanna et al. reported pyloric metaplasia in 15% of cases and intestinal metaplasia in 16% [38]. Increased gallbladder wall thickness was also observed, correlating with the increased incidence of metaplasia. Given the close interaction between chronic inflammation and neoplastic processes, it is reasonable to assume that changes associated with chronic cholecystitis may trigger early metaplastic changes in the gallbladder epithelium, which could progress to dysplastic lesions and eventually neoplastic transformations. The metaplasia-dysplasia-cancer sequence has been well established in the development of gallbladder carcinoma, with a rough estimate of about 10 years for the appearance of neoplastic features [39].

Therefore, early surgical intervention, particularly in younger patients, could prevent this catastrophic scenario. Currently, the standard practice is to perform a cholecystectomy in symptomatic patients. This intervention typically eliminates the long-term risk of gallbladder cancer before the appearance of precursor lesions. However, it is essential to note that the clinical presentation of calculous cholecystitis is more evident in cases of macrolithiasis, where obstructive symptoms are more pronounced, while microlithiasis can remain subclinical for a longer period [40].

The overall prevalence of metaplastic changes in the gallbladder epithelium in our study aligns with previous reports, with metaplastic changes being more common in patients with microlithiasis. This raises an important question about the role of preventive cholecystectomy in this patient group, especially after the accidental detection of microlithiasis in abdominal imaging, particularly in younger patients. Previous studies have reported a positive correlation between the number, weight, and size of gallstones and the presence of concomitant metaplastic and dysplastic lesions in the gallbladder. Metaplastic changes in the context of chronic cholecystitis are not uncommon, especially in patients with microlithiasis. Microlithiasis appears to be more frequently associated with concurrent metaplastic and dysplastic lesions compared to macrolithiasis, which could theoretically be attributed to the long-term but more diffuse negative impact on gallbladder epithelial cells. Macroscopically, these early changes appear to be correlated with the presence of a thicker gallbladder wall, suggesting a possible role for routine ultrasonographic evaluation to stratify high-risk patients, particularly in the absence of clinical symptoms that would prompt earlier cholecystectomy [41].

In our study, patients with metaplasia predominantly exhibited pyloric metaplasia, followed by osseous metaplasia, with no cases of intestinal metaplasia. This difference in the type of metaplasia observed in our study suggests a distinct distribution compared to other studies, which have reported more frequent cases of intestinal metaplasia. These variations may be explained by the fact that pyloric and osseous metaplasia are often associated with chronic inflammation of the gallbladder, particularly in cases of chronic cholecystitis. Pyloric metaplasia may appear as a response of the gallbladder epithelium to inflammation, while osseous metaplasia, though rare, may occur in the context of severe and prolonged inflammatory processes. In contrast, intestinal metaplasia is more commonly linked to long-standing inflammation or conditions that promote intestinal epithelial cell proliferation, such as chronic inflammatory bowel disease, persistent bacterial infection, or microbiological imbalances. Each patient may have a different genetic predisposition to develop certain types of metaplasia. This variability may contribute to the prevalence of specific metaplastic forms based on environmental conditions and the patient’s medical history. It is important to note that, given the relatively small sample size of this study, the results may be influenced by the selection of participants and the specific characteristics of the studied group. Differences in the type of metaplasia could also reflect demographic and clinical peculiarities of the studied population. Some studies have noted that pyloric metaplasia is more frequently observed in the context of chronic cholecystitis or other biliary disorders, due to the specific characteristics of local inflammation. Osseous metaplasia, although rare, may result from an adaptive response or a mineralization and calcification imbalance in the context of chronic inflammation or prolonged pathological processes.

As mentioned earlier, the cases of metaplasia observed in our study consisted of pyloric and osseous metaplasia, with no cases of intestinal-type metaplasia identified. Another important observation in this study is that all cases of metaplasia were found in patients with a history of chronic cholecystitis, suggesting that chronic inflammation of the gallbladder may play a significant role in the development of this histopathological condition. This association was statistically significant (*p* = 0.005), indicating that patients with chronic cholecystitis may be more susceptible to developing metaplasia. This finding highlights the importance of closely monitoring patients with chronic cholecystitis, as metaplasia is often a precursor to more severe lesions, including malignant conditions. The analysis of risk factors, laboratory parameters, and tumor markers suggests that gallbladder metaplasia is a complex condition influenced by a variety of factors, including comorbidities such as obesity and hypertension, as well as changes in biological and biochemical parameters. These findings may help improve diagnostic and treatment strategies for patients with metaplasia, emphasizing the importance of personalized management for each patient.

Recent pan-cancer studies have increasingly emphasized the significant role of the tumor immune microenvironment (TIME) in tumor initiation, progression, and therapeutic response. The TIME consists of a complex network of immune cells, cytokines, and signaling molecules that interact with tumor cells and influence both disease evolution and treatment outcomes. In a previous pan-cancer analysis, it was hypothesized that estrogen-related receptor gamma (ESRRG) might be positively associated with lymph node metastasis in cholangiocarcinoma (CHOL). Although a tendency toward higher ESRRG expression in more advanced CHOL cases was observed, the relatively limited number of CHOL samples available in the analyzed database prevented the association from reaching statistical significance. To further investigate this potential relationship, immunohistochemical analysis of ESRRG expression was subsequently performed in gallbladder cancer specimens and in samples of cholecystitis. The results demonstrated a significantly higher frequency of ESRRG positivity in gallbladder carcinoma compared with inflammatory gallbladder tissue, with markedly increased expression levels in malignant samples. Furthermore, elevated ESRRG expression was significantly associated with advanced TNM stage, deeper tumor invasion, and the presence of lymph node metastases. Survival analysis indicated that positive ESRRG expression correlated with shorter progression-free survival, while the association with overall survival showed a similar trend but did not reach statistical significance. These findings suggest that ESRRG may play a particularly relevant role in mechanisms related to tumor progression, recurrence, and metastatic spread. Multivariate survival analyses further identified ESRRG expression as an independent prognostic factor and predictor of recurrence in patients with gallbladder cancer [42].

Interest in circulating biomarkers for the diagnosis of gallbladder cancer (GBC) has increased considerably in recent years. Earlier investigations evaluated classical tumor markers, while more recent studies have applied proteomic technologies to identify novel candidates. More recently, in 2023, Baichan et al. employed liquid chromatography–mass spectrometry to evaluate tissue and plasma samples and identified nine proteins with altered expression in both compartments, including APOA1, APOA2, RET4, TTR, HEMO, HBB, HBA, PIGR, and APOE [43]. Other investigations have focused on protein signatures detected at different tumor stages, including those derived from extracellular vesicles and circulating plasma proteins. In particular, a 2021 study identified 29 proteins contained in plasma-derived extracellular vesicles that were uniquely dysregulated in early-stage GBC, with three candidates—NT5E, ANPEP, and MME—subsequently validated as potential biomarkers. Despite these advances, most earlier studies relied primarily on conventional statistical comparisons between cancer and control groups [44]. Such frequency-based approaches may be susceptible to increased false-positive rates due to multiple testing and often identify associations rather than biomarkers with strong predictive value. Consequently, Bayesian model-based machine learning approaches have been proposed as a complementary strategy, as they can manage high-dimensional datasets and prioritize biomarker selection based on predictive performance.

Although progress has been made in identifying candidate biomarkers, the etiology and molecular mechanisms underlying GBC remain incompletely understood. The most consistently recognized risk factors include gallstones and chronic cholecystitis, while additional contributors such as advanced age, female sex, chronic infections with Salmonella or Helicobacter species, and primary sclerosing cholangitis have also been implicated. Proteomic analyses have begun to reveal several proteins that may be involved in the biological processes driving GBC development and progression. For example, alterations in PAHX may affect peroxisomal lipid metabolism, a pathway increasingly associated with biliary tract tumorigenesis. DEPP has been linked to oxidative stress responses, which may contribute to the pro-oxidative microenvironment that promotes malignant transformation. Other molecules, such as CRISP2, VEGF sR2, HRG, and DKK2, are involved in immune regulation, angiogenesis, and Wnt signaling, all of which are key processes in tumor progression and immune evasion. Furthermore, several additional proteins—including SERPINA1, ANXA3, COL6A1, PRTN3, and KRT18—have been associated with GBC diagnosis through both statistical analyses and machine learning approaches. Additional candidates identified through computational models include KRT1, ANXA2, PPIB, CSF1, and ERBB3. Some of these markers have previously been linked to lymph node metastasis or have been proposed as therapeutic targets in biliary tract malignancies. Overall, these findings highlight the complex molecular landscape of GBC and support the ongoing search for novel circulating biomarkers that may improve early detection and preoperative diagnosis beyond tumor markers such as CA19-9, CA242, CA125, or CEA [45].

Our study has several limitations that should be acknowledged. First, it is a single-center, non-randomized observational study with a relatively small sample size, which may limit the generalizability of the findings to broader populations. Second, the follow-up period was relatively short (six months), restricting the assessment of long-term oncological outcomes, including recurrence and survival. Third, the study included only patients with operable and radiologically suspected gallbladder lesions, which may introduce selection bias and limit applicability to advanced or inoperable disease. Additionally, although we evaluated multiple tumor markers and inflammatory parameters, the lack of an extended proteomic or molecular panel may have limited the ability to fully characterize the complex biological interactions underlying gallbladder pathology. Furthermore, potential confounding factors related to comorbidities, such as cardiometabolic and renal dysfunction, may have influenced the observed associations. Finally, the absence of external validation further limits the robustness of our findings. Therefore, larger, multicenter studies with longer follow-up periods and more comprehensive biomarker profiling are warranted to confirm our results and improve their clinical applicability.

Nonetheless, our results were in line with previous research and showed that the mean level of CA 19-9 was significantly greater than the level of CEA, indicating that CA 19-9 is typically more increased in adenocarcinoma cases. This pattern suggests that CA 19-9 might be a more accurate marker for identifying this kind of malignancy. Additionally, the range of CA 19-9 levels was narrower than that of CEA, indicating less variation in the concentration of the marker between patients. This lower variability advocate that CA 19-9 levels are more uniform between cancer patients. CEA values, on the other hand, showed a larger range and a higher standard deviation, indicating more variation in CEA concentrations within the same patient group. This fluctuation may have an effect on CEA’s consistency as a diagnostic tool, making it less reliable than CA 19-9. The data confirms that CA 19-9 has greater stability and less volatility than CEA, in addition to having a higher average level in cases of adenocarcinoma. These results highlight CA 19-9’s potential as a more accurate biomarker for adenocarcinoma. Because of its higher mean level and lower variability, it is a good option to increase the precision of adenocarcinoma diagnostic tests and monitoring, which could result in improved patient outcomes and management.

The tumor markers carbohydrate antigen 19-9 (CA 19-9) and carcinoembryonic antigen (CEA) are essential for the diagnosis and treatment of numerous malignancies, including those that impact the digestive tract. When evaluating tumors including pancreatic, bile duct, colorecta, and stomach cancers, these markers are extremely important. While higher total bilirubin levels, for example, may have an impact on CA 19-9’s efficacy in predicting tumor resectability in gallbladder cancer (GBC) cases, its real diagnostic capability may be obscured.

The potential of CA 19-9 in detecting GBC is demonstrated by its increased sensitivity, but the integration of numerous markers could improve diagnostic accuracy and clinical utility even more.

Larger sample sizes and longer follow-up times should be the main goals of future studies in order to confirm these results and improve the application of tumor markers in patient outcomes.

Ultimately, these advancements may lead to enriched early detection, more effective treatment regimens and increased rates of patient survival for those with gallbladder cancer.

A limitation of this study is the absence of a control group with similar clinical and radiological findings but benign histopathology. Although all patients were initially suspected of having GBC, some had non-neoplastic conditions, which may also influence biochemical parameters. Future studies should include matched controls to better assess the diagnostic value of these markers.

## 5. Conclusions

According to our study, CA 19-9 displays higher mean levels than CEA, suggesting that it has a higher sensitivity for adenocarcinoma detection. CA 19-9 levels’ narrower range and lower fluctuation lend credence to their status as a stable and trustworthy biomarker. CEA levels recorded a wider range and more fluctuation, which would have limited its diagnostic consistency. This supports the idea that CA 19-9, which has greater average levels and improved stability, is a more reliable marker for adenocarcinoma and a good option for enhancing diagnostic precision.

Although using CA 19-9 and CEA alone has its benefits, combining these markers could provide a more thorough method of detecting and treating GBC.

Even more, gallbladder pathology should be considered within the broader spectrum of cardiometabolic disease, where inflammation, metabolic imbalance, and tumor biology intersect, offering new opportunities for integrated diagnostic and therapeutic approaches.

## Figures and Tables

**Table 1:** Demographic characteristics.

Number of Patients—60 Parameter	Range	Mean	Median
Age	28–85 years	64.5 years	64 years
Hemoglobin (Hb)	8.90–16.10 g/dL	13.16 g/dL	13.20 g/dL
Hematocrit (Ht)	28–46%	39.96%	39.30%
Leucocytes	4450–26,000 cells/µL	11,743 cells/µL	11,800 cells/µL
Platelets	156,000–477,000 cells/µL	251,383 cells/µL	252,000 cells/µL
TGO (AST)	11–176 U/L	23.93 U/L	18 U/L
TGP (ALT)	13–70 U/L	22.85 U/L	22 U/L
Creatinine (cr)	0.51–1.56 mg/dL	1.08 mg/dL	1.01 mg/dL
Sodium (Na)	134–147 mmol/L	139.2 mmol/L	139 mmol/L
Potassium (K)	3.07–5.40 mmol/L	3.73 mmol/L	3.70 mmol/L
CRP	0.29–384 mg/L	27.77 mg/L	18 mg/L
Total Bilirubin	0.51–176 mg/dL	19.36 mg/dL	14.00 mg/dL
FA (Alkaline Phosphatase)	0.67–107.11 U/L	23.41 U/L	18.00 U/L
COVID-19	Negative–Positive	20% Positive	-
CA 19-9	2.1–588.9 U/mL	51.76 U/mL	11.2 U/mL
CEA	0.29–147.85 ng/mL	7.33 ng/mL	2.4 ng/mL
AFP (alpha fetoprotein)	0.29–70 ng/mL	5.98 ng/mL	2.21 ng/mL

**Table 2:** Relationship between tumor indicators and both benign and malignant gallbladder disease.

Group	CA19-9 Mean (U/mL)	CA19-9 Median (U/mL)	CA19-9 Range (U/mL)	CA19-9 Std Dev (U/mL)	CEA Mean (ng/mL)	CEA Median (ng/mL)	CEA Range (ng/mL)
Non-Adenocarcinoma	30.0	30.0	4	2.83	38.5	38.5	13
Adenocarcinoma	84.29	11.2	623	149.5	1.15	1.00	31.06

**Table 3:** Multivariate Analysis of Predictive Factors for CA19-9 Levels in Patients with Adenocarcinoma (Histopathological Examination).

Model	R	R^2^	Adjusted R^2^	Std. Error	F	*p*	Predictors
1	0.153	0.023	0.012	122.52830	1.968	0.164	Age
2	0.681	0.463	0.450	91.41007	66.333	0.001	Age, CRP
3	0.935	0.874	0.869	44.62990	259.798	0.001	Age, CRP, CEA
4	0.994	0.987	0.987	14.12832	719.290	0.001	Age, CRP, CEA, CA125
5	0.994	0.988	0.987	14.21171	0.076	0.784	Age, CRP, CEA, CA125, AFP
6	0.994	0.988	0.987	14.12429	1.969	0.165	Age, CRP, CEA, CA125, AFP, Amylase

**Table 4:** Multivariate Analysis of CA19-9 Levels in Patients with Metaplasia: Influence of Predictors.

Model	R	R Square	Adjusted R	Std. Error	F	*p*	Predictors
1	0.224	0.050	0.039	180.74119	4.289	0.042	Age
2	0.380	0.145	0.123	172.59977	8.822	0.004	Age, CRP
3	0.991	0.983	0.982	24.63849	3846.926	0.001	Age, CRP, CEA
4	0.998	0.996	0.996	11.66644	274.354	0.001	Age, CRP, CEA, CA125
5	0.998	0.996	0.996	11.59598	1.951	0.167	Age, CRP, CEA, CA125, AFP
6	0.998	0.996	0.996	11.37909	3.963	0.050	Age, CRP, CEA, CA125, AFP, Amylase

**Table 5:** Multivariate Analysis of Factors Influencing CRP Levels in Patients with Adenocarcinoma.

Model	R	R Square	Adjusted R	Std. Error	F	*p*	Predictors
1	0.196	0.039	0.027	93.65	3.285	0.074	Age
2	0.349	0.122	0.100	90.06	7.674	0.007	Age, Hb
3	0.410	0.168	0.137	88.21	4.422	0.039	Age, Hb, HT
4	0.418	0.175	0.133	88.38	0.691	0.408	Age, Hb, HT, Amylase
5	0.718	0.515	0.484	68.18	54.761	0.001	Age, Hb, HT, Amylase, CA19-9
6	0.751	0.564	0.530	65.07	8.626	0.004	Age, Hb, HT, Amylase, CA19-9, CEA
7	0.752	0.565	0.525	65.41	0.203	0.653	Age, Hb, HT, Amylase, CA19-9, CEA, AFP
8	0.752	0.566	0.520	65.78	0.144	0.706	Age, Hb, HT, Amylase, CA19-9, CEA, AFP, CA125

**Table 6:** Multivariate Analysis of Factors Influencing CRP Levels in Patients with Metaplasia.

Model	R	R^2^	Adjusted R^2^	Standard Error	F	*p*	Predictors
1	0.196	0.039	0.027	93.65	3.285	0.074	Age
2	0.349	0.122	0.100	90.06	7.674	0.007	Age, Hb
3	0.410	0.168	0.137	88.21	4.422	0.039	Age, Hb, HT
4	0.418	0.175	0.133	88.38	0.691	0.408	Age, Hb, HT, Amylase
5	0.718	0.515	0.484	68.18	54.761	0.001	Age, Hb, HT, Amylase, CA19-9
6	0.751	0.564	0.530	65.07	8.626	0.004	Age, Hb, HT, Amylase, CA19-9, CEA
7	0.752	0.565	0.525	65.41	0.203	0.653	Age, Hb, HT, Amylase, CA19-9, CEA, AFP
8	0.752	0.566	0.520	65.78	0.144	0.706	Age, Hb, HT, Amylase, CA19-9, CEA, AFP, CA125

## Data Availability

The raw data supporting the conclusions of this article will be made available by the authors on request.

## References

[1] Rosato V., Gómez-Rubio P., Molina-Montes E., Márquez M., Löhr M., O’Rorke M., Michalski C.W., Molero X., Farré A., Perea J. (2021). Gallbladder disease and pancreatic cancer risk: A multicentric case-control European study. Eur. J. Cancer Prev..

[2] Bhoge A., Khandeparkar S.G.S., Joshi A.R., Gogate B., Kulkarni M.M., Bhayekar P. (2017). Immunohistochemical study of MUC1 and MUC5AC expression in gall bladder lesions. J. Clin. Diagn. Res..

[3] Barreto S.G., Dutt A., Chaudhary A. (2014). A genetic model for gallbladder carcinogenesis and its dissemination. Ann. Oncol..

[4] Bojan A., Pricop C., Ciocoiu M., Vladeanu M.C., Bararu Bojan I., Badulescu O.V., Badescu M.C., Plesoianu C.E., Halitchi D.I., Foia L.G. (2024). Environmental and Metabolic Risk Factors Linked to Gallbladder Dysplasia. Metabolites.

[5] Jain K., Mohapatra T., Das P., Misra M.C., Gupta S.D., Ghosh M., Kabra M., Bansal V.K., Kumar S., Sreenivas V. (2014). Sequential occurrence of preneoplastic lesions and accumulation of loss of heterozygosity in patients with gallbladder stones suggest causal association with gallbladder cancer. Ann. Surg..

[6] Sharma A., Sharma K.L., Gupta A., Yadav A., Kumar A. (2017). Gallbladder cancer epidemiology, pathogenesis and molecular genetics: Recent update. World J. Gastroenterol..

[7] Bojan A., Foia L.G., Vladeanu M.C., Bararu Bojan I., Plesoianu C., Plesoianu A., Pricop C. (2022). Understanding the mechanisms of gallbladder lesions: A systematic review. Exp. Ther. Med..

[8] Mochidome N., Koga Y., Ohishi Y., Miyazaki T., Matsuda R., Yamada Y., Aishima S., Nakamura M., Oda Y. (2021). Prognostic implications of the coexisting precursor lesion types in invasive gallbladder cancer. Hum. Pathol..

[9] He C.Z., Zhang K.H., Li Q., Liu X.H., Hong Y., Lv N.H. (2013). Combined use of AFP, CEA, CA125 and CA 19-9 improves the sensitivity for the diagnosis of gastric cancer. BMC Gastroenterol..

[10] Wang Y.F., Feng F.L., Zhao X.H., Ye Z.X., Zeng H.P., Li Z., Jiang X.Q., Peng Z.H. (2014). Combined detection tumour markers for diagnosis and prognosis of gallbladder cancer. World J. Gastroenterol..

[11] Zhang D., Yu M., Xu T., Xiong B. (2013). Predictive value of serum CEA, CA19-9 and CA125 in diagnosis of colorectal liver metastasis in Chinese population. Hepatogastroenterology.

[12] Ma W., Li W., Wang J., Wu R., Liu C., Feng F., Jiang X. (2020). The Clinical Role of Preoperative Serum CA19-9 and Carcinoembryonic Antigen (CEA) Levels in Evaluating the Resectability of Advanced Gallbladder Cancer. Med. Sci. Monit..

[13] Liu F., Wang J.K., Ma W.J., Yang Q., Hu H.J., Li F.Y. (2019). Clinical value of preoperative CA19-9 levels in evaluating resectability of gallbladder carcinoma. ANZ J. Surg..

[14] Hickman L., Contreras C. (2019). Gallbladder cancer: Diagnosis, surgical management, and adjuvant therapies. Surg. Clin. N. Am..

[15] Marrelli D., Caruso S., Pedrazzani C., Neri A., Fernandes E., Marini M., Pinto E., Roviello F. (2009). CA19-9 serum levels in obstructive jaundice: Clinical value in benign and malignant conditions. Am. J. Surg..

[16] Rawla P., Sunkara T., Thandra K.C., Barsouk A. (2019). Epidemiology of gallbladder cancer. Clin. Exp. Hepatol..

[17] Balakrishnan A., Barmpounakis P., Demiris N., Andersson B., Brañes A., de Aretxabala X., Eilard M.S., Gibbs P., Harper S.J.F., Huguet E.L. (2025). Assessment of nodal staging and risk factors for nodal involvement in gallbladder cancer. BJS Open.

[18] Yu T., Yu H., Cai X. (2014). Preoperative prediction of survival in resectable gallbladder cancer by a combined utilization of CA 19-9 and carcinoembryonic antigen. Chin. Med. J..

[19] Wen Z., Si A., Yang J., Yang P., Yang X., Liu H., Yan X., Li W., Zhang B. (2017). Elevation of CA19–9 and CEA is associated with a poor prognosis in patients with resectable gallbladder carcinoma. HPB.

[20] Agrawal S., Gupta A., Gupta S., Goyal B., Siddeek R.A.T., Rajput D., Chauhan U., Kishore S., Gupta M., Kant R. (2020). Role of carbohydrate antigen 19-9, carcinoembryonic antigen, and carbohydrate antigen 125 as the predictors of resectability and survival in the patients of Carcinoma Gall Bladder. J. Carcinog..

[21] Sachan A., Saluja S.S., Nekarakanti P.K., Nimisha Mahajan B., Nag H.H., Mishra P.K. (2020). Raised CA19-9 and CEA have prognostic relevance in gallbladder carcinoma. BMC Cancer.

[22] Zhou X. (2022). Meta-analysis of the diagnostic performance of serum carbohydrate antigen 19-9 for the detection of gallbladder cancer. Int. J. Biol. Markers.

[23] Sinha S.R., Prakash P., Singh R.K., Sinha D.K. (2022). Assessment of tumour markers CA 19-9, CEA, CA 125, and CA 242 for the early diagnosis and prognosis prediction of gallbladder cancer. World J. Gastrointest. Surg..

[24] Shukla V.K., Gurubachan Sharma D., Dixit V.K. (2006). Diagnostic value of serum CA242, CA 19-9, CA 15-3 and CA 125 in patients with carcinoma of the gallbladder. Trop. Gastroenterol..

[25] Ramos-Font C., Gómez-Rio M., Rodríguez-Fernández A., Jiménez-Heffernan A., Sánchez R.S., Llamas-Elvira J.M. (2014). Ability of FDG-PET/CT in the detection of gallbladder cancer. J. Surg. Oncol..

[26] Ramphal W., Boeding J.R.E., van Iwaarden M., Schreinemakers J.M.J., Rutten H.J.T., Crolla R.M.P.H., Gobardhan P.D. (2019). Serum carcinoembryonic antigen to predict recurrence in the follow-up of patients with colorectal cancer. Int. J. Biol. Markers.

[27] Mishra P.K., Saluja S.S., Prithiviraj N., Varshney V., Goel N., Patil N. (2017). Predictors of curative resection and long term survival of gall bladder cancer—A retrospective analysis. Am. J. Surg..

[28] Strom B.L., Maislin G., West S.L., Atkinson B., Herlyn M., Saul S., Rodriguez-Martinez H.A., Rios-Dalenz J., Iliopoulos D., Soloway R.D. (1990). Serum CEA and CA 19-9: Potential future diagnostic or screening tests for gallbladder cancer?. Int. J. Cancer.

[29] Shukla P.J., Neve R., Barreto S.G., Hawaldar R., Nadkarni M.S., Mohandas K.M., Shrikhande S.V. (2008). A new scoring system for gallbladder cancer (aiding treatment algorithm): An analysis of 335 patients. Ann. Surg. Oncol..

[30] Palakollu V.N., Veera Manohara Reddy Y., Shekh M.I., Vattikuti S.V.P., Shim J., Karpoormath R. (2024). Electrochemical immunosensing of tumor markers. Clin. Chim. Acta.

[31] Ghosh M., Sakhuja P., Singh S., Agarwal A.K. (2013). p53 and beta-catenin expression in gallbladder tissues and correlation with tumour progression in gallbladder cancer. Saudi J. Gastroenterol..

[32] Eil R., Hansen P.D., Cassera M., Orloff S.L., Sheppard B.C., Diggs B., Billingsley K.G. (2013). Bile duct involvement portends poor prognosis in resected gallbladder carcinoma. Gastrointest. Cancer Res..

[33] Hundal R., Shaffer E.A. (2014). Gallbladder cancer: Epidemiology and outcome. Clin. Epidemiol..

[34] Huang J., Lucero-Prisno D.E., Zhang L., Xu W., Wong S.H., Ng S.C., Wong M.C.S. (2023). Updated epidemiology of gastrointestinal cancers in East Asia. Nat. Rev. Gastroenterol. Hepatol..

[35] Rawal N., Hariprasad G., Bandyopadhyay S., Ranjan Dash N., Kumar S., Das P., Dey S., Ahmad Khan M., Ranjan A., Chopra A. (2024). Molecular biomarkers involved in the progression of gallbladder inflammatory lesions to invasive cancer: A proteomic approach. Biomol. Biomed..

[36] Narang S., Goyal P., Bal M.S., Bandlish U., Goyal S. (2014). Gall stones size, number, biochemical analysis and lipidogram-an association with gall bladder cancer: A study of 200 cases. Int. J. Cancer Ther. Oncol..

[37] Seretis C., Lagoudianakis E., Gemenetzis G., Seretis F., Pappas A., Gourgiotis S. (2014). Metaplastic changes in chronic cholecystitis: Implications for early diagnosis and surgical intervention to prevent the gallbladder metaplasia-dysplasia-carcinoma sequence. J. Clin. Med. Res..

[38] Khanna R., Chansuria R., Kumar M., Shukla H.S. (2006). Histological changes in gallbladder due to stone disease. Indian J. Surg..

[39] Sharma S., Walia B.S., Randhawa M., Sharma A., Dugg P., Pannu J.S. (2023). Histopathological changes in gall bladder mucosa in relation to the number, and size of gallstones, and analysis of the findings in the context of age distribution of the patients: A perspective. Ann. Hepatobiliary Pancreat. Surg..

[40] Shafique M.S., Ahmad R., Ahmad S.H., Hassan S.W., Khan J.S. (2018). Gallstones in young population. J. Ulutas Med..

[41] Dq W., De C., Mc C. (2009). Biliary lipids and cholesterol gallstone disease. J. Lipid Res..

[42] Gong W., Wen S., Chen Y., Wu F., Yang M., Sun P., Guo X., Li M., Chen D., Zhao H. (2025). Deciphering ERR family genes as prognostic and immunological biomarkers through pan-cancer analysis with validation in gallbladder cancer. Front. Oncol..

[43] Baichan P., Naicker P., Augustine T.N., Smith M., Candy G., Devar J., Nweke E.E. (2023). Proteomic analysis identifies dysregulated proteins and associated molecular pathways in a cohort of gallbladder cancer patients of African ancestry. Clin. Proteom..

[44] Priya R., Jain V., Akhtar J., Chauhan G., Sakhuja P., Goyal S., Agarwal A.K., Javed A., Jain A.P., Polisetty R.V. (2021). Plasma-derived candidate biomarkers for detection of gallbladder carcinoma. Sci. Rep..

[45] Nouairia G., Cornillet M., Jansson H., Bergquist A., Sparrelid E. (2025). Towards precision medicine strategies using plasma proteomic profiling for suspected gallbladder cancer: A pilot study. JHEP Rep..

